# “We are champions”: an investigation on how cancer support group propels patient advocacy, voices, education, mentorship, and support

**DOI:** 10.1007/s00520-025-10203-7

**Published:** 2025-12-06

**Authors:** Runcie C. W. Chidebe, Swat Kasa Gimba, Agha A. Agha, Oluwatobiloba D. Oguntoyinbo, Leigh Leibel, Banwo Faridah Mobolanle, Ahmed Danquah, Mide Agbola, Chinwendu V. Igboekulie, Aman Shrestha, Chioma Nwakanma-Akanno, Ifeoma J. Okoye, Maria Chidi C. Onyedibe

**Affiliations:** 1 Project PINK BLUE – Health and Psychological Trust Centre, Abuja, Nigeria; 2 Scripps Gerontology Center, Miami University, Oxford, OH USA; 3 Department of Sociology and Gerontology, Miami University, Oxford, OH USA; 4 Network of People Impacted By Cancer in Nigeria, Abuja, Nigeria; 5 Department of Social Work, University of Nigeria, Nsukka, Nigeria; 6 Tennessee Oncology, Nashville, TN USA; 7 Division of Hematology/Oncology, Columbia University Irving Medical Center, New York, NY USA; 8 School of Nursing, University of Pittsburgh, Pittsburgh, USA; 9 Department of Chemistry, Tufts University, Medford, MA USA; 10 Division of Gerontology, Department of Epidemiology and Public Health, University of Maryland School of Medicine, Baltimore, MD USA; 11 Smile With Me Foundation, Lagos, Nigeria; 12 University of Nigeria, Nsukka Centre of Excellence for Clinical Trials, Enugu, Nigeria; 13 Department of Psychology, University of Nigeria, Nsukka, Nigeria; 14 Faculty of Epidemiology and Population Health, London, School of Hygiene and Tropical Medicine , London, UK

**Keywords:** Supportive care, Patient engagement, Peer support groups, Patient and public involvement, Patient-centered care

## Abstract

**Purpose:**

With over 127,000 incidents and 79,000 deaths, cancer is now officially declared a notifiable disease of public interest in Nigeria. Most cancer control efforts have targeted prevention, diagnosis, and treatment, often overlooking supportive care, survivorship, and patient-centered aspects of care. This study investigated survivors’ experiences with cancer support groups (CSG), exploring their activities, effectiveness, challenges, and benefits to address these critical gaps.

**Methods:**

Participants (*N* = 14) who were cancer survivors and members of a CSG were recruited. Data collection was conducted through interviews, and the data were analyzed using both inductive and deductive thematic analysis.

**Results:**

Five key themes were identified: “[coming] together, gives us a voice” (Patients’ Voice); “[we] are dying from this cancer … who will speak out for us?” (Patient Advocacy); “I feel better [that] I have more understanding of the disease” (Patient Education); “I have a mentor and … I mentor” (Peer Mentor); and “support that we draw from each other” (Peer Support). The categories illustrated the facilitators, barriers, benefits, activities, and optimism in CSGs. The results showed that CSGs could propel patient advocacy, give a voice to survivors, provide education, mentorship, and supportive care to members.

**Conclusions:**

The findings highlight the role of CSGs in supportive care and survivorship. Establishing CSGs at cancer centers may promote patient advocacy and education and reduce abscondment. Nigeria’s National Cancer Control Plan should prioritize and fund supportive care. Low- and middle-income countries should focus on engaging and collaborating with cancer patients to advance their cancer control efforts.

**Supplementary Information:**

The online version contains supplementary material available at 10.1007/s00520-025-10203-7.

## Introduction

Cancer is a major public health issue in Africa, with one in thirteen people at risk of developing cancer before the age of 75 years, and one in nine faces the risk of dying from the disease in 2022 [1]. Between 2018 and 2022, more than 2.6 million people in Africa lived with cancer [2], and it is estimated that 1.4 million new cancer cases and 0.9 million related deaths will be recorded by 2030 [3]. In Nigeria, over 127,763 new cases of cancer and 79,542 deaths were recorded in this population of over 230 million people in 2022 [3]. In 2024, Dr. Tunji Alausa, the Minister of State for the Federal Ministry of Health and Social Welfare, declared that cancer is now officially a notifiable disease of public interest in Nigeria—which means that private and public healthcare institutions are now obliged to report all cancer cases to the National Institute on Cancer Research and Treatment (NICRAT). The government also launched a $212 million (NGN97 billion) National Strategic Cancer Control Plan (2023–2027) with eight strategic goals and priority areas of action, including prevention, diagnosis/treatment, supply chain management, hospice, advocacy, data management, governance, and survivorship [4]. All the priority areas of the strategic cancer plan were budgeted for each year; only survivorship care has a zero budget for all 5 years. On this premise, survivorship care may not be adequately addressed in government policies, and there is a corresponding lack of emphasis on supportive care, including patient-centered data, psychosocial support, patient advocacy, education, and cancer support groups (CSGs).

Advocacy is increasingly recognized as a crucial mechanism for addressing the growing cancer burden in many countries. According to VeneKlasen and Miller [5], “Advocacy is a continuous process which leads to positive change in attitudes, behavior, and relationships within the family, workplace, and community, and state and society, i.e., all social institutions”. On the other hand, patient advocacy refers to the active involvement of individuals with lived experiences, either as cancer patients, survivors, or informal caregivers of cancer patients, in seeking policy changes and promoting broader goals for themselves and their communities—including improving access to resources and supporting research advancements [6]. Patient advocacy is crucial in cancer control, as it provides cancer patients and survivors with opportunities to have a voice in their care and treatment, as well as the care of others or their community [7]. Yet, advocacy, cancer advocacy, and patient advocacy are understudied and largely misunderstood or used in place of patient awareness in Nigeria. For clarity, cancer awareness refers to the state and process of publicizing and increasing knowledge and information about cancer within a community and for the public, whereas advocacy aims to change policies, attitudes, and practices.

Furthermore, patient and public involvement (i.e., patient engagement) in research, intervention, treatment, and medicine development is well documented as essential for shaping clinical trials, cancer prevention and control programs, and ensuring that treatment and research are patient-centered [8]. Shared decision-making (SDM) is a collaborative process where clinicians and patients exchange information, consider available options, and agree on a care plan that reflects both medical expertise and patient values [9]. SDM emphasizes mutual respect, patient education, and culturally sensitive communication, ensuring that decisions are informed, inclusive, and centered on what matters most to the patient. However, SDM remains limited as patients are often largely passive in their care in Nigeria and most parts of Africa. Without incorporating patients’ perspectives, voices, and experiences, there is often a disconnect between the designed interventions, treatment, and the actual needs, values, acceptance, and concerns of the patients [10]. This gap can contribute to disparities in cancer incidence and mortality rates. Hence, engaging patients and giving them a voice has the potential to ensure that health services, government plans, and cancer research reflect the real-world experiences of the patients [8, 10]. However, patient voices and engagement have been underrepresented in Nigeria’s oncology literature.

It is well documented that CSGs play a vital role in creating patient awareness and leading advocacy for patient-centered care, fostering a supportive community, and ensuring that the experiences and needs of those affected by cancer are addressed in healthcare policies and research initiatives [11, 12]. For instance, a study of advocacy movements led by civil society organizations in 23 low- and middle-income countries (LMICs) across Eastern Europe, East and Southern Africa, Central Asia, and Latin America reveals that cancer survivors and patients serving as advocates are invaluable in advancing cancer control efforts [13]. However, patient advocacy is still in its nascent stages in Nigeria and Africa, where cancer patients and survivors rarely speak up or have the platform to speak up for themselves or others in the cancer journey. CSGs have been explored as a crucial platform for patient advocacy in countries with limited resources. However, the nature and the process have remained unknown in Nigeria and across Africa.

In recent years, CSGs have emerged in certain parts of Nigeria. In 2017, Runcie C.W. Chidebe founded the Abuja Breast Cancer Support Group (ABC-SG), and later founded the Network of People Impacted by Cancer in Nigeria (NePICiN) in 2019 to become an umbrella support group for cancer patients [14]. Several other patient advocates and organizations have also established CSGs, and a few studies have investigated their importance to patients and survivors. For instance, Esan et al. [15] reported that 37% of 90 cancer patients utilize belonging to a CSG as a coping strategy. CSGs have been shown to improve patients’ quality of life by reducing distress levels and enhancing their functional and emotional well-being [16]. Despite the importance of CSGs, previous studies exploring CSGs in Nigeria have overlooked several key aspects, including what CSGs do, how they operate, who participates, and the benefits of belonging to CSGs from the patients’ perspectives. The establishment of CSGs, activities, facilitators, and barriers associated with membership of CSGs are largely unknown. Hence, the present study aims to investigate survivors’ experiences with CSGs, exploring their activities, effectiveness, challenges, and benefits, to address these critical gaps. Specifically, we investigated the following questions: *(i) What are cancer survivors’ perspectives on patient advocacy and cancer support groups? (ii) What activities are conducted within cancer support groups? (iii) What factors facilitate the effectiveness of cancer support groups? (iv) What challenges do cancer support groups face? (v) What are the perceived benefits of belonging to cancer support groups?* We argue that answering these questions may offer the data and evidence needed for patient advocates, non-governmental organizations, and facilities to establish CSGs in this population.

## Method

### Study design

This qualitative study followed social constructivist paradigms, which is an interpretative framework that argues that individuals seek understanding of the world they find themselves in and gain knowledge through social interaction and shared meanings [17]. Based on the above, phenomenology was adopted as the most suited approach for describing the lived experiences, the meaning attached to the phenomenon of interest, and to grasp the very nature of the thing (i.e., belonging to a CSG and the lived experience of cancer). Specifically, transcendental phenomenology was applied; therefore, we focused strictly on the participants’ interpretations of their lived experiences and the descriptions of meanings [18]. Hence, we bracketed out our experiences, collected data from several cancer patients, analyzed the data, and examined the findings through the voices of the participants [18]. To apply bracketing thoroughly, we also avoided using any theoretical or empirical interpretation; instead, we focused on the phenomenon and the essence of the experience. This study was reported using the Consolidated criteria for Reporting Qualitative Research (COREQ, see Online Supplementary for the checklist) [19].

### Participants and recruitment

Participants (*N* = *14*), aged 38 years and above and who have been clinically diagnosed with cancer, were purposively recruited through the ABC-SG and NePICiN. The inclusion criteria include: being a cancer survivor for at least the past 2 years, a member of any CSG in Nigeria for the past 6 months, and having started or completed any cancer treatment. Cancer survivors who are less than 2 years into treatment and are not members of any CSG were excluded because they may not have sufficient lived experience of the phenomenon of interest; see Table 1 for the demographic and clinical characteristics of the participants. The participants’ recruitment was led by two female cancer survivors, who were leaders of the ABC-SG and NePICiN; they announced the “call for participation for the study on belonging to CSG” to all the cancer survivors in the CSGs at the monthly meeting and also through the WhatsApp group (i.e., an online group chat). Interested cancer survivors contacted the two leaders, and they were added to a WhatsApp group where patient information sheets and informed consent forms were uploaded for all the study participants. Working with leaders is an effective patient engagement strategy in research because participants are more familiar with the leaders than with the researchers. For the participants who read the forms and wanted to participate, two authors—AAA (a male lecturer of social work/PhD candidate) and OCN (a female physician)—scheduled and conducted the interviews at the preferred patients’ time and day. Using WhatsApp for recruitment was helpful (i.e., easy for the survivors to use) in our setting; however, it still presented some privacy risks, such as visibility of metadata, even with end-to-end encryption. To address these risks, we limited the data shared within the group and used direct messages for any exchanges involving identifiable information. All the cancer survivors were encouraged to respond to the telephone interviews at a convenient place where only they would be present to enable them to speak freely without interruption. All participants who were recruited participated in the study. Each participant provided oral informed consent, and they received N5,000 ($3.5) for their call card. Oral consent was considered appropriate since the interviews were conducted over the phone, making written consent logistically difficult. This study was performed in line with the principles of the Declaration of Helsinki and was approved by the National Hospital Abuja Health Research Ethics Committee.

**Table 1:** Demographic and clinical characteristics of participants

Pseudonyms	Age	Sex	Marital status	Type of cancer	Stages of diagnosis	Year of diagnosis	Year of joining the CSG
Ada	*	F	*	BC	II	2010	2017
Vida	47	*	M	BC	II	2020	2022
Fiona	*	F	*	BC	IV	2016	2022
Sola	*	M	*	HL	*	*	2022
Obi	40	F	S	BC	II	2012	2017
Ngozi	54	F	M	BC	II	2018	2020
Efe	45	F	S	BC	I	2018	2020
Fumi	50	F	M	BC	I	2018	2019
Tope	49	F	M	BC	II	2013	2020
Ola	38	F	M	BC	III	2016	2018
Annie	59	F	M	BC	*	2018	2019
Nora	*	F	M	BC	III	2017	2018
Temi	38	F	D	BC	II	2017	2019
Milly	39	F	M	BC	I	2022	2022

*BC* breast cancer, *HL* Hodgkin’s lymphoma

*Survivors did notdisclose

### Data collection

A semi-structured interview was conducted using the telephone. In transcendental phenomenological studies, the semi-structured interview is recommended because it provides the researchers a platform to ask participants broad and open-ended questions about their lived experiences with the phenomenon and follow up with additional questions to gain a better understanding of the meaning, contexts, and common experiences of the participants [17]. An interview guide was developed by the authors based on existing literature on CSG and their experiences in cancer survivorship. Some of the interview questions were: “What has been your experience as a member of the cancer support group?,” “What has been the benefit of the cancer support group?,” “What attracted you to the cancer support group?,” “What is your experience with peer support or mentorship?,” “How do think cancer support group can be improved to care for cancer patients in Nigeria?,” and “What is your experience with cancer diagnosis and treatment?.” The interview guide was shared with a CSG leader to gather feedback and was also piloted with two participants before the actual interview. After the pilot, no questions were changed or removed. AAA and OCN have relational and practice skills that are useful in the facilitation of a semi-structured interview [20]; hence, they built rapport and asked probing questions on shared decision making, patient–doctor communication, CSG activities, and sustainable ways of improving cancer treatment and care in Nigeria. Participants also provided demographic and clinical characteristics such as date of diagnosis, cancer stage, date of joining CSG, age, sex, and marital status; however, some survivors declined to respond to some of the questions. All participants were asked not to mention their names; hence, they were given pseudonyms during data analysis. The interviews were conducted in English, audio recorded using a mobile phone, and lasted for 60 min or more. The transcripts were manually transcribed verbatim by a local research associate, cleaned up by the research team, and uploaded to Dedoose (a data management platform). While data saturation is often cited as a common reason for determining sample size, this is not always applicable in many qualitative health studies; therefore, some qualitative scholars emphasize that the characteristics of the research and theoretical perspectives are crucial [21–23]. In our study, the information power model was used to justify the sample size and data collection, rather than relying solely on saturation. The information power model indicates that the more information participants provide, the more relevant it is to the study’s aim, and consequently, a smaller sample size is needed [24]. Therefore, the number of participants deemed necessary is appropriate for this study.

### Data analysis

We conducted two analyses, inductive and deductive thematic analysis as described by Braun and Clarke [25]. With this method of analysis, we were interested in both describing and unraveling the realities of cancer survivors and their experiences of belonging and participating in CSG. In this study, themes were identified based on information that captured important meaning in relation to the research goal and the experiences of cancer survivors [26]. All the themes in this study were data-driven and described the overarching meaningful essence across the participants’ experiences, responses, and perspectives through inductive thematic analysis [25, 26]. On the other hand, categories were defined as a collection of similar responses sorted into the same place using deductive thematic analysis [26]. Hence, deductive thematic analysis was used to develop the *categories*, while the *themes* were identified using inductive thematic analysis as follows:

### (i) Inductive thematic analysis

*First*, one of the researchers (RCWC, a male patient advocate and oncology researcher) cleaned the transcripts again, removing all possible identifiers, and stored the files. RCWC, CVI (a female graduate student), and AD (a physician assistant and MSc student) all familiarized themselves with the transcripts. Two separate analyses were done by CVI and RCWC/AD. *Second*, the researchers started coding inductively while reading and re-reading the transcripts. The initially generated codes were discussed during a team research meeting. *Third*, using the coded transcripts, the researchers sorted, collated, and extracted potential themes. The identified themes were arranged in a table, and the team determined if they truly represented the meaningful essence of the participants’ perspectives. *Fourth*, the research team started reviewing themes and sub-themes to check if each theme had sufficient quotes to support the data and tested for referential adequacy. *Fifth*, the researchers started defining each theme. All the theme names were inductive, data-driven, and developed from the data. *Sixth*, at this stage, the entire theme, its definition, and supportive quotes were written logically and concisely. The research team met to discuss the results, and a consensus was achieved on all the themes.

### (ii) Deductive thematic analysis

*First*, we developed a set of codes based on our research objectives (stated in the introduction). *Second*, RCWC and AD read and re-read all the transcripts. *Third*, categories are coded on Dedoose. Similar responses from the transcripts that reflect each research objective were extracted and placed in a column of a table. *Fourth*, a similar process was applied to all transcripts on a case-by-case basis. *Fifth*, the final categories were developed with supporting quotes, in some cases renamed, defined, and arranged in a table that reflected the research questions. All the researchers had weekly meetings for 3 months during the analysis to discuss the results. A consensus was reached for all the categories; see Table 3 for the categories. Based on the inductive and deductive findings, a Ten-Step Framework for Creating and Sustaining a Support Group was developed; see Fig. 3. Additionally, expert reviews were sought from seven specialists in patient engagement, cancer survivorship, supportive care, and cancer control from Africa, America, Australia, Europe, and Asia via a survey; see the Online Supplementary for the survey results.

### Reflexivity

Throughout the study, all authors remained aware of our positionalities and how they could influence recruitment, data collection, analysis, and interpretation. The lead researcher (RCWC), a patient advocate and founder of the CSGs, was intentionally excluded from recruitment, data collection, and transcription to reduce potential bias. SKG, LL, and MA are cancer survivors, with SKG and MA also members of the CSG; however, they were not involved in participant recruitment. Other authors (AAA, ODO, FMB, AD, CVI, AS, CAN, IJO, MCCO) had no prior relationship with participants. We collaboratively discussed the themes and categories and challenged our interpretations to ensure the results accurately reflected the participants’ voices before proceeding to trustworthiness.

### Trustworthiness

The trustworthiness of the results was established using member checking and the thematic analysis strategy. We shared the manuscript with two cancer survivors and conducted a focus group on Zoom (an online meeting platform) for the survivors to review and comment on their own data, ensuring that the interpretations, meanings, and descriptions were credible and accurate. After the member checking, the survivors confirmed that the results accurately reflected the participants’ responses; therefore, no further changes were made.

## Results

The demographic and clinical characteristics of the participants are presented in Table 1. The results are shown as inductive themes below, with a summary presented in Table 2, while the deductive categories are displayed in Table 3. The analytical linkage illustrating the relationship between the research questions, inductive themes, and deductive categories is described in Table 4.

**Table 2:** Summary of themes from the inductive thematic analysis

*Themes*	*Inductive themes (sources from participants’ quotes)*
Patients’ voice	“[Coming] together, gives us a Voice”
Patient advocacy	“[We] are dying from this cancer … who will speak out for us?”
Patient education	“I feel better [that] I have more understanding of the disease”
Peer mentor	“I have a mentor and … I mentor”
Peer support	“Support that we draw from each other”

**Table 3:** Categories from the deductive thematic analysis

*Categories*	*Definition*	*Source*
***Facilitators of cancer support group***		
“Need to be understood”	Refers to how the CSG provides a platform for establishing relationships and acceptance of each other	“The need for bonding. Because when you do not meet with these people, you just feel all alone. In fact, you feel you are all alone. So, that need for bonding, that need to be understood, that need to be encouraged and [also] encourage others” **Efe**
“I usually feel very lonely and I talk to myself a lot.”	The CSG helps patients share their challenges and change their perception of the situation	“I usually feel very lonely, and I talk to myself a lot, I ask myself a lot of questions, and sometimes I cannot even find answers to any of the questions I ask myself. But after joining the group, I now met other members there who have similar problems, we share our problems, and you have this particular thing sometimes you want to relate it back to the group, and relate it to the treatment you have taken.” **Tope**
“Feel relieved”	Refers to how listening to each other’s stories improves patients’ general mental wellbeing	“So sometimes, it feels that emotional need and mental needs because when you are there, you feel your case is worst but you hear other people share their stories, and you feel you have been helped mentally because there are many things you have bottled up in your head but you hear other people talking about it and all, so you feel relieved.” **Efe**
**“**The teaching…in that WhatsApp [group] is also helping me.**”**	The information sharing on the CSG WhatsApp group has been very helpful and useful to the patients	“I have not been attending the meeting but the teaching and other things people are sending in that WhatsApp [group] is also helping me. So, I have been benefiting a lot from the group.” **Fumi**
“We are the living proof. We are the survivors, We are the Champions”	Refers to the belief that the CSG is changing its viewpoint that cancer is not a death sentence	“Not at all because they are family and I am also looking forward to meeting new members because most people tell you once you have cancer, it is a death sentence but no, we are the living proof. We are the survivors, we are the champions” **Tope**
***Barriers faced by cancer support groups***		
Lack of funding for the cancer support group	This refers to the financial constraints experienced by survivors due to the lack of support faced by the CSG, limiting its ability to function and provide sustained support	“[cancer] support group is a body of its own. It is not owned by the government, it is an NGO…If we have government and individuals who are ready to support the group, I think any cancer patient who needs support will get it from the group.” **Tope**
Limited employment opportunities for cancer survivors	This is the battle for employment among cancer survivors	“We really have a very long way to go in that our group. First of all, we really need funds because most of us are still on treatment, and most of us do not have work. Even me, I don’t have work.” **Vida**
Inadequate access to psychotherapy	This refers to the challenges in getting psychotherapy as part of cancer treatments	“Ok, that psychotherapy, if they can actually extend it, because I think that now if you want to go, you need to book an appointment first. So, let it be something that the psychologist will be there like a permanent job or even if it cannot be every day, let it be like three times a week so we can have more access,” **Ola**
Poor acceptance of the disease	This refers to the difficulties experienced by some patients in accepting their diagnosis and beginning treatments	“I see a future where anyone diagnosed with the disease can easily identify him or herself with the group. A lot of the patients are still living in the denial stage, they do not want to say it so that people won’t get to know.” **Efe**
“Cancer needs money”	This refers to the costs associated with the treatment of cancer	“I go with my transport and my time, so it tells you that this is also my concern, not just project pink and blue. The treatment is expensive. For example, now I am an applicant, I don’t work anymore and you ask me to call you, if not that you are the one that called me, where do I get the credit to even call you like this? Do you understand? Secondly, I still maintain my diet and it is expensive. Imagine someone that does not work, we need support from the government because cancer needs money,” **Fiona**
**Benefits of belonging to cancer support group**		
“This support group always tell you where to go” [peer navigation]	This refers to how cancer patients provide peer-to-peer navigation information on treatment and care	“The benefit is that many people do not know where to go when they have this cancer. And this support group always tell you where to go and how to follow it, and even counsel you because many people think that if you have cancer, that is a death sentence.” **Annie**
Access to free and discounted mammogram	Refers to how the CSG provides free and discounted mammograms to the members	“The first mammogram I did is from there, that one was free because it was from there that I knew it was taking place. They just selected the first ten people, and I was among the first ten. The second one, they said we pay half, which I paid and benefited from it” **Fumi**
“Gives me hope and courage”	New patients believed that seeing patients who have been post-treatment for long gives them hope and courage of survivorship	“The benefit is at least it gives me hope and courage. I saw people that have been in this thing for years and they are surviving so it gives me the hope that I too, I am a survivor.” **Vida**
“I was helpless…they paid for my radiation”	Refers to how the CSG provides financial support for treatment to the members	“Within those 9 months, I was helpless, I did not know what to do, but when I joined the support group, I opened up to them and they gave me funds for my radiotherapy, they gave me six hundred thousand (N600, 000) for my radio, they paid for my radiation” **Nora**
“We normally pay half.”	Defines how the CSG supports patients with part payment for mammograms and other care	“And another thing is that most times when I go for checkup, they send me for mammogram. I have done it through the support group. You know most of the time when they are doing their promo and other things, they always consider us, and even if we are going to pay, we normally pay half.” **Fumi**
“Psychological part of it is the most important to me”	The view that mental health support is one of the most important benefits of the CSG	“And of course there are also benefits of having meetings and speaking to doctors, people give us gifts but the psychological part of it is the most important to me, and I am sure a lot of people will agree to that too.” **Sola**
**Activities of cancer support group**		
“We sing, dance and say nice things to each other”	This refers to how patients celebrate each other in CSG	“So, it helps us emotionally, mentally because when go for these meetings, we have some sessions where we sing and dance and say some nice things to each other. **Efe**
“Every month we meet once”	This refers to the frequency of meeting in the CSG	“Every month we meet once and we have a doctor that will come and talk with us, share ideas and how best we can do things and live, that is it.” **Fiona**
“We had a series of events…We have photoshoot”	They expressed making memories together in the CSG by taking group photos	“we normally have events that we are talk to. In October, we had a series of events to celebrate the breast cancer awareness month, we have photoshoot sometimes, a lot of events. So, at these events, we meet with each other, we talk, share our experiences, have fun together, and do a lot of things together.” **Efe**
“Share your experience”	This refers to how CSG encourages cancer patients to open up and discuss their personal journeys	“It has been very interesting, at least it gives one opportunity to meet other cancer patients. When you meet other cancer patients, you can share your experiences with the, you can talk about your feelings and find out from them if they are feeling the same thing” **Ada**
“Looking for mastectomy bra for almost a year”	This refers to unavailability of prosthesis for cancer survivors	“But I think one thing that is overlooked, in fact I have already prepared a post about it for social media is a gap that can be filled. And this is around prosthetics and other cancer things for cancer survivors. I remember I was looking for a mastectomy bra for almost a year. If you go to the US for example, there are mastectomy stores, for any laundry store, if they do not have a mastectomy bra, they can tell you where to get it.” **Sola**
“social gathering together”	This refers to how meeting at the CSG fosters social activities	“think it is the bonding, the joy of seeing each other outside the hospital, and sometimes we do some little social gathering together.” **Ada**
“Share my personal experiences, feelings and ways of coping with the disease”	This refers to how the CSG provides a platform for sharing lived experiences and coping strategies	“Being a member, I have had the opportunity to bond with other cancer patients during our meetings and some other events we attend, the support group affords me the opportunity to share my personal experiences, feelings and ways of coping with the disease.” **Efe**
“Series of event to celebrate breast cancer awareness month”	Refers to hosting different events to commemorate cancer global days	“In October, we had a series of events to celebrate the breast cancer awareness month, we have photoshoot sometimes, a lot of events. So, at these events, we meet with each other, we talk, share our experiences, have fun together, and do a lot of things together” **Efe**
“All the encouragement that I need”	The CSG is helping the survivors encourage each other	“Just like I told you, I am making friends already, the encouragement is there, even if it is not direct, telling other people “you can do it, you can pull through” and all that is enough for me. But behind the scene I am getting all the encouragement I need from other people, so why would I leave the group” **Milly**
“It gives me satisfaction helping another person to survive”	Patients derive satisfaction in helping other patients through the CSG	“The strength I draw from there, the encouragement they give to other people. Though I am the quiet type in the group, I have been participating. One day, someone just asked for money in the group and I quietly paid for the blood. Because it gives me satisfaction helping another person to survive.” **20**
“See the psychologist”	Refers to having access to a psychologist who provides mental support and wellbeing	“Every month, they bring a psychologist and you can ask questions because that psychologist is very important for cancer patients. You can also book an appointment and see the psychologist on some available dates.” **Ola**
“Manage my stress and pains”	The CSG educates patients on how to manage pain	“My stress level has actually reduced. After joining that group, I learnt a whole lot of things about how to manage my stress and pains.”** Tope**
“Leaving means going into solitude”	This acknowledges the consequences they might incur leaving the CSG	“Will you consider leaving the group? P: Leaving! no, no, no. I: Why? P: Leaving means going into solitude, I will be alone again.” **Efe**
**Optimism within the CSG**		
“Understand myself more”	This refers to how CSG helps patients accept their diagnosis and begin to actively participate in their care and treatment	“And I also understand myself more now, I try to listen to my body because after the oncologist might have said one or two things, it has really helped me to [better] understand myself as a patient.” **Tope**
“Not shy away”	This category refers to the stigma associated with cancer and how the CSG is positioned to reduce stigma in society	“I see future where those diagnosed with cancer will not shy away or not wanting to be associated with such a group.” **Efe**
“The group is the reason that I am still moving”	Refers to patients networking with patients through CSG helps give life to patients	“It really benefitted me. If there is anyone that benefitted in that cancer support group, I feel I am the one because so many things. That group is the reason that I am still moving on.” **Efe**
“Cancer is not a death sentence”	This refers to the realization of hope after the diagnosis of cancer	“I felt as if life had ended when I was diagnosed but when I met them, that was when life started. I learnt that cancer is not a death sentence, and that with cancer you can still do so many things, as long as you are taking your treatment.” **Obi**
“I started feeling alive”	How seeing other survivors at the CSG helps others perceive themselves as being alive	“When I was told I have cancer, I did not even eat, I was looking dry because I thought there was no need of eating, no need of living, let that death come. But when I joined that group, I started feeling alive. If you see me today, I don’t even look like I have cancer.” **Obi**
“Encourage you to take chemo”	This refers to the view that the CSG survivors stick to orthodox treatment and adhere to the care	“They will encourage you because you are with people who have the experience. They will encourage you to take chemo, and you begin to get yourself. They will tell you different things to do.” **Obi**
“Men also have breast cancer”	The CSG provides opportunities for cancer knowledge, increasing awareness among members	“So, I feel it is my responsibility to know everything knowable about breast cancer and surviving breast cancer. For example, if I was not constantly talking about breast cancer, I would not know that men also have breast cancer and because of that, to me no knowledge is wasted.” **Sola**
“I asked if I would be alive if I had one breast”	The CSG provides answers to the patients’ questions, providing reassurance	“I was even given the option of doing hormonal therapy but considering everything, I asked if I would be alive if I had one breast, because that was the confirmation that I needed. So I just went for the mastectomy because it gave me the most peace of mind of all the options.” **Sola**
“Hope and courage”	This implies that the CSG serves as a beacon of hope for newbies	“The benefit is at least it gives me hope and courage. I saw people that have been in this thing for years and they are surviving so it gives me the hope that I too, I am a survivor.”** Vida**
“Donate… blood”	The CSG eases the hurdles of blood donation	“There was a lady that needed five pints of blood, some of us had to be donating small small. So, we really need help, and if they go through the support group, they are on the right channel. Anyone, because we know where to go to, we know ourselves.” **Vida**

**Table 4:** Analytical linkage between the inductive themes and the deductive categories

Research questions	Inductive themes	Deductive categories	Interpretive links
RQ1: What are cancer survivors’ perspectives on patient advocacy and cancer support groups?	Themes 1 and 2: “[Coming] together, gives us a voice” & “[We] are dying from this cancer… who will speak out for us?”	Facilitators to belonging to cancer support groups	While the themes detailed the participants’ views on patient advocacy and how the CSG gives them a voice that they do not have in their oncology clinics, the deductive categories showed that these features of a CSG are important to the participants
RQ2: What activities are conducted within cancer support groups?	Themes 3, 4, and 5: “I feel better [that] I have more understanding of the disease,” “I have a mentor and … I mentor” & “support that we draw from each other.”	CSG activities: monthly meetings, sing, dance, photoshoot, donation, see the psychologist, etc	The categories contrast the themes in some regards. The categories provided a list of diverse activities that are more entertaining and fun-related. Themes 3, 4, and 5 provided robust data that are somewhat more serious. The functionality of the CSG hinges on hosting both serious conversations and fun-related events that are meaningful to cancer survivors
RQ3: What factors facilitate the effectiveness of cancer support groups	Participants’ excerpts: “need to be understood”? “the teaching…in that WhatsApp [group] is also helping me.”	The CSG offers a platform for sharing information, building relationships, listening to each other, and changing how they view cancer	These opportunities to connect are important in facilitating the effectiveness of the CSG. While the CSG meets monthly, the use of a WhatsApp group is an innovative platform that keeps survivors connected in their fight against cancer
RQ 4: What challenges do cancer support groups face?	Theme 1 and 2: “[coming] together, gives us a voice” & “[we] are dying from this cancer… who will speak out for us?”	Lack of funds, inadequate access to psychotherapy, poor acceptance of the disease, and lack of employment	Themes 1 and 2, which emphasize patient voice and advocacy, support the categories that enumerate the challenges of the survivors. Although CSG provided platforms for survivors to speak up and advocate for themselves, the CSG is not in a position to implement policy changes that can improve care. Hence, the CSG needs to create and partner on hosting high-level policy meetings that make the changes possible
*RQ5: What are the perceived benefits of belonging to cancer support groups*	*Theme 4:* “I have a mentor and … I mentor”	The CSG functions as a peer navigation outlet, providing access to free and discounted mammograms and financial patient navigation	While the theme presented a unique form of supportive care between the survivors, the categories specified the approaches of the mentorship. These benefits are meaningful to the survivors, especially in a country where access to healthcare services is limited and often requires out-of-pocket payments

### Theme 1: “[coming] together, gives us a voice” (patients’ voice)

This theme refers to the participants’ perspectives on the importance of giving cancer patients opportunities and platforms to speak up and share their wishes, desires, feelings, concerns, and opinions concerning their disease and their healthcare team. The participants narrated that the oncology healthcare system was created in such a way that cancer patients accept everything the system provides and rarely question or seek clarification on issues relating to their disease and the healthcare system. Most participants asserted that patient–doctor communication is usually one-directional—where the doctor is speaking and the patients are listening; however, the CSG provided a bi-directional platform where the patients can meet with the doctors and speak freely about their disease and healthcare system. For instance, Ada said:

To me, [the cancer support group]… gives us a voice. You know, when you go to the clinic, you do not have that kind of voice. You just listen to the doctor, and the doctor is the one talking. But during the support group meetings, the oncologist comes, and it is sweet talking to the oncologist.

Promoting patients’ voices is beneficial to the oncology healthcare system, the patient, other patients, and the public. “I see a whole lot of people … becoming more outspoken, and it is helping them deal with stigma,” said Sola. They are becoming more daring, sharing their experiences, helping others, and trying to create a ripple effect for others who are yet to join. So, I think these small things will keep on improving, so I am seeing very positive things about us. Right now, the results are small, but I see it growing because of the resolve of those in charge,” said Temi. For Fiona, she said, “I will always want to be a cancer ambassador anywhere; you will know that you can survive it if you keep to the rules, especially the advice that come from doctors. But the Project PINK BLUE, I give them kudos, they are doing very well.”

Many participants described that the CSG provided an opportunity to share their views on oncology care and treatment, which empowered them, provided a sense of belonging, and boosted each others’ confidence in shared clinical treatment decisions. Most of the participants believe that by giving cancer patients a voice in their care, the oncology healthcare system ameliorates their fears, manages their stress, and builds trust in the care and treatment. On this premise, many of the participants argued that joining a CSG should be integrated into the oncology care and treatment of the hospitals. Sola argued, “So, your mind has to be ready for the stress involved before they take you through the treatment. So, I definitely agree that joining a support group should be part of the treatment. I agree hundred and one percent.”

### Theme 2: “[we] are dying from this cancer… who will speak out for us?” (patient advocacy)

This theme describes the participants’ understanding of the power of patient advocacy in propelling the accessibility, availability, and affordability of cancer treatment in the population. Participants share the view that cancer patients can mobilize themselves through the CSG, articulate their challenges, and approach high-level policymakers at different government levels to bring the needed policy change that can improve the lives of cancer patients and bring about better outcomes. “Advocacy is very important…We have been doing it. We go to the government, we advocate for people to hear our voice because cancer treatment is not a child’s play” Tope**.** Most of the participants believe that if cancer patients come together and drive policy advocacy, the government will listen and act on their needs and challenges. One of the participants shared:

We can gather and go to the government…they can do for us like [they did for] HIV because AIDS has free treatment now… If I am to meet any House of Representatives to complain for them to listen to cancer patients… I am ready. Now, some people [cancer patients] don’t even have money to go for their treatment. [they] don’t have work. Let us join together and push so our cancer patients can get treatment. Annie

Many of the participants argue that many cancer patients are dying because they cannot afford the life-saving medications prescribed to them by their doctors. Temi said, “The biggest challenge of a cancer patient is money.” Hence, there is a need for the patients to drive policy advocacy that can bring about timely access to care and treatment. Annie supports Tope’s view with her statement on the impact of cancer. She said: “Women are dying from this cancer… who will speak out for us? That is why this cancer support group should speak out, write a letter or meet all these government… this is what I think we do to go forward to achieve our goals” Annie.

While some participants focused on self-advocacy for change in access to cancer care and treatment, Temi, Annie, and many others emphasized advocacy for others since they had already survived cancer. In Tope’s words, “we go out there, we advocate, they should hear our voice, they should see the survivors, people have survived it. If not for we that have survived it, then for the people that will come after us, they should get support throughout their treatment” Tope. Based on this, the participants presented two strands of patient advocacy: self-advocacy and advocacy for others.

### Theme 3: “I feel better [that] I have more understanding of the disease” (patient education)

Participants believe they need to understand the disease well to make informed choices throughout their diagnosis, treatment, and survivorship. While most of the participants acknowledged learning about their cancer diagnosis from their doctors, they elaborated that they had burning questions and worries that they could not ask their doctors at the hospital due to limited consulting time and the fear of offending the doctors and healthcare team. The participants elaborated that the CSG is a major source of patient education for both new and old cancer patients and a safe, open, and welcoming space for addressing questions, promoting discussions freely, and knowledge sharing from different points of view without fear of offending the doctor or healthcare teams. “Many things we [patients] are afraid of, they [CSG] brought all these psychology people, especially our oncology doctors, to tell us many things we should do. What we cannot even ask them in the hospital, we can fully ask them there and they tell us.” Annie. For the participants, having a space where they can freely ask questions about their cancer journey and get answers helps them feel better. Tope shared her views:

Joining the group has helped me…I usually feel very lonely and I talk to myself a lot, I ask myself a lot of questions and sometimes I cannot even find answers to any of the questions I ask myself. But after joining the group, I now met other members there who have similar problems, we share our problems, and you have this particular thing sometimes you want to relate it back to the group, and relate it to the treatment you have taken… I feel better [that] I have more understanding of the disease.

Understanding the disease is crucial for most of the participants, as many of the participants narrated that they were never educated about many parts of their disease and what would happen after many treatment regimens. For instance, Sola exclaimed angrily, “It was at the support group that I learnt that even after mastectomy, breast cancer can reoccur.” While many participants assert that the CSG provides a platform for patient education, other participants (Obi, Nora, Efe, and Tope) argue that the CSG also provides patients with education that helps to transition new patients into accepting chemotherapy, surgery, and other forms of treatment and care as prescribed by the doctors. A newly diagnosed participant shared how meeting another cancer patient inspired her to proceed with her treatment. Nora said:

When I was diagnosed, I knew nothing about cancer, I had not heard about it or seen anybody suffering cancer and I was like where will I start from. But through the help of the [cancer] support group, I got people that have survived it, people that had gone through the treatment, they encouraged me, they helped me, and so many things my brother, Even the things I am using like prosthetics bra, they do give us.

Just like Nora, Efe also shared how the education that she received at the CSG meeting helped her to believe in chemotherapy. Efe narrated, “I was still contemplating; should I go in for chemotherapy whatever. So, I made a friend that day and she told me she just finished her chemotherapy and surgery and all that. I was like all the stories I have been hearing about chemotherapy that have been making me scared of presenting myself for treatment, if this person can go through it and withstand the pains and all that, what is the big deal?”.

Additionally, most participants narrated that they learn new information about their disease: “Every day, new things are coming and the person that had the experience will share it and we benefit…we learn everyday” said Fumi**.** The monthly CSG meetings feature diverse oncology sessions (i.e., learning about recurrence, symptomatic management) led by clinical oncologists and group psychotherapy sessions led by a clinical psychologist. Ada said, “…there have been psychological sessions we learn from and it kind of balances some emotional trauma we do have.” Although the majority of the participants alluded to the patients’ education from professionals such as clinical oncologists, psychologists, nurses, and other health professionals, some participants argue that there is a lot of education that the patients themselves learn from each other (i.e., peer education). “Cancer is not something you just sit at home and be doing it all alone. You need to have experienced people [i.e., cancer patients]. So, every meeting, we learn about it [cancer]. I achieve so many different things every day of our meeting.” said Vida.

### Theme 4: “I have a mentor and … I mentor” (peer mentorship)

Participants feel more heard, understood, and valued by having another cancer survivor whom they can call or talk to at any point in time regarding their disease or treatment, including their choice of medications or psychological support. “So, sometimes in the morning or midnight, somebody will call me and say, I am going through this; what am I supposed to do? So we mentor each other, we share experiences; this is what we are supposed to do, and this is what we are not supposed to do” Tope. This is the role of mentorship in the CSG: helping participants manage their disease with a more patient-centered peer-to-peer approach and addressing individual needs beyond the spectrum of the scheduled CSG monthly meetings. This also served as an opportunity for all survivors to be mentored and provide mentorship to other patients to help them with any kind of information when needed. Tope shared her experience:

I had pains in my right heel, I went to the hospital and nobody was able to tell me what the problem was, when I go and complain, they only give me pain relief and when I take the pain relief, after twelve hours, the pain comes back twice the pain I was feeling before I took the medication. So I started making research on my own and somebody told me it is this, I found out that was the pain I was going through and I found solution to it. When I spoke to my mentor, he was like I should go to the internet and search, I did and it was right.

For some participants, these shared mentorship roles became an avenue for developing good friendships that some now refer to as “sisters.” Ada shared, “you suddenly become like a blood sister because from the support group, we have some people that we mentor, especially we that have been there for a long time… and even if you feel you cannot do it, you can direct them to where they will have the help. Recognizing the benefits of mentorship, the doctors even recommend mentorship opportunities through the CSG to newly diagnosed patients. “Some doctors… are the ones that will even recommend to a patient and tell them we have such groups” Ada. Thus, the mentorship process breeds a sense of belongingness and brings about satisfaction, making everyone feel responsible for taking care of other patients to achieve the desired care throughout their survival journey. “Also, I have a mentor, and I also have people I mentor, and that gives me a whole lot of hope and support because I feel I have somebody I can run to and ask question and I feel satisfied,” said Tope.

### Theme 5: “support that we draw from each other” (peer support)

Peer support is what cancer patients refer to when they provide or receive meaningful, symptomatic, and psychological support from other patients who may have little or more experience. Achieving the necessary support to survive cancer is not an individual battle, but rather requires long-term interdependency on each other throughout the process. According to Fumi, “it’s a lifetime thing. You cannot just…say that you want to do everything on your own; that is why it is called a support group. You need to support somebody with your lifestyle and all the experiences you have.

I went to the hospital and I saw a lady, she was just crying, it was like they diagnosed her newly. She was crying, she thought she was going to die. I encouraged her but I had to call somebody that has been on the group before me and directed her to the person. After everything, she thanked me, she is fine and she is doing her treatment. When you see other people that have gone through it so many years and they are alive, when they talk to you, you will feel better. Nora

This happened to be the experience of a newly diagnosed patient who drew strength and support from a peer, another patient. This emphasizes the immense support that the CSG provides, including knowing other patients. One said, “Well, personally, I draw strength from there, knowing there are people who have been through the same thing…. Just seeing them happy, knowing they are still moving on, it gives me joy” Milly. Another patient shared:

I think the benefit is the support that we draw from each other. You have this feeling that you are not alone, that other people are going through the same thing you are going through, and get to talk to them. You may talk to your caregiver and they will just say sorry because they don’t understand you. But if you are talking to another cancer patient or survivor, the person understands what you are saying and how you are feeling. Temi

Despite the benefits mentioned, demonstrating the crucial role of peer support during the cancer journey, a patient who had a terrible experience with another patient [a prior survivor] and other healthcare workers elicited the need for getting support from the “right” peers/persons. She shared the following after being asked by the interviewer whether she would advise people to join the CSG: “It’s just that peer support. It is something I did not enjoy earlier. That 2017/2019, it was just me and one other woman I met that instead of using her journey to strengthen me, she used it to scare me.” Temi.

### Ten-step framework for creating and sustaining a support group

This ten-step framework provides a replicable, patient-centered model for establishing, maintaining, and evaluating support groups. Divided into three phases—initiation, formation, and sustainability—it highlights emotional connections, survivor leadership, and institutional integration to ensure lasting impact. The ten-step framework is a cycle of continuous processes that extend throughout the lifespan of the support group. Therefore, the framework can be used by patients, survivors, advocates, family caregivers, clinicians, non-governmental organizations, and healthcare facilities to create a cancer support group (CSG) or a support group for other disease areas. The framework is also useful in evaluating the progress of a support group and assessing its viability and sustainability. Support groups that incorporate all the steps have the potential to continue recruiting and engaging patients while achieving long-term sustainability.

## Discussion

**Fig. 1:**
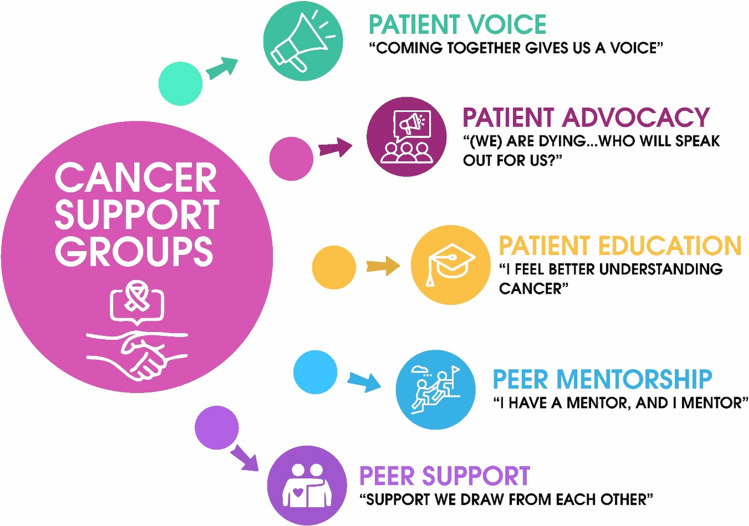
Thematic connections in relation to cancer support groups. The figure five interrelated themes that emerge from participation in cancer support groups. Central to the figure is the role of support groups as a hub fostering: (i) Patient Voice, (ii) Patient Advocacy, (iii) Patient Education, (iv) Peer Mentorship, and (v) Peer Support. Together, these demonstrate how cancer support groups enhance both individual and collective patient experiences

CSGs, patient engagement, advocacy, and supportive care in cancer play a crucial role in addressing patients’ needs beyond disease-specific treatments. However, data and literature on this area of oncology are scarce in Nigeria. Therefore, this study investigated survivors’ experiences with CSGs, exploring their activities, effectiveness, challenges, and benefits to address these critical gaps. Our results indicate that CSGs serve as a source of supportive care, uplifting patient voices, and enhancing communication, providing opportunities for patient advocacy, increasing patient education, facilitating peer mentorship among patients, and fostering peer support among survivors, see Fig. 1 and Table 4. To our knowledge, this is the first study exploring the activities of CSGs and their operations in Nigeria and Africa. The findings will be valuable for cancer institutions, nonprofit organizations, leaders, and policymakers in their efforts to enhance supportive care with innovative and data-driven strategies.

### Shared decision-making in the treatment of cancer patients

The vulnerability of cancer patients often leads to their alienation from decision-making regarding their care plans and treatment choices. The main finding of this study revealed that cancer patients need a voice in their care, and the CSG provides this voice, enabling them to participate in the decision-making process concerning their treatment. Patients gain strength through sharing and cooperation with other patients to make their wishes known and to request that their wishes be respected regarding their treatment plans and choices. This is a form of rights that patients exercise both at home and in the hospital. Allowing cancer patients to be involved in decision-making will help achieve inclusive care plans and improved outcomes and survivorship. This is corroborated by Søndergaard et al. [27], who reported that engaging patients in shared decision-making (SDM) reduced anxiety associated with cancer recurrence and decision regrets.

The key components of SDM in cancer treatment include educating patients about the disease, clarifying treatment options, fostering trust, and encouraging collaboration [28]. However, our findings show that patients are seldom educated about their disease or given a voice in their treatment decisions. Consequently, no form of SDM is accessible to this population. Through the CSG activities, patients gain valuable insights that empower them to engage actively in the treatment decision-making process. One of the participants said, “It was at the support group that I learnt that even after mastectomy, breast cancer can recur.” While this is an excerpt from one participant, many others recounted a similar lack of understanding regarding the disease and education beyond treatment. Engaging patients in shared decision-making by the oncology team is a best practice, as evidenced by studies from Spronk et al. [29], Kehl et al. [30], and Kashaf and McGill [31]. By implication, our findings highlight the need for oncology professionals to “talk with” and not “talk to” patients about their treatment and care plans. Our findings emphasize that oncology care and treatment plans should be discussed “with patients,” providing information in a bi-directional manner, enabling patients to ask questions and receive answers that empower them to make treatment decisions that are meaningful to them as individuals and not merely about treating the disease. Although the availability of the clinical oncology workforce [32], patient literacy, and cultural barriers [33] could pose challenges to the implementation of SDM in this setting, it is important to highlight that SDM improves patient outcomes, enhances patient engagement, and addresses patient-specific needs [28, 34].

### Peer mentorship: a novel supportive care approach for patients guiding patients

A central finding of this study revealed a unique and novel approach by patients utilizing peer mentorship to support and guide other patients in this CSG. Our findings indicate that peer mentoring offers one-on-one personalized intervention between survivors or more experienced patients and newly diagnosed patients. This is a novel approach that is least reported in the literature yet holds great significance for cancer survivorship. Typically, some members of a support group can feel lost in a crowd due to their inability to fully engage in the group’s activities for various reasons. However, through the peer mentorship arrangement established by this CSG, no one is left behind, as everyone is recognized and paired to oversee and closely monitor their partners. The paired members have the responsibility of following up on one another, and everyone is expected to report on their progress and how each is faring in their cancer care journey during CSG meetings. These reports are articulated by the group and assessed to determine if any intervention is necessary to support any member. Limited studies highlight the role of peer mentorship in supportive care and cancer survivorship. Where it was reported, it was not presented as a unique and innovative mechanism driven by the patients themselves.

For instance, the Ovarian Cancer Research Alliance’s Woman to Woman (W2W) program and Peer Support Providers (PSP) studies are among the few that recognized the impact of peer mentorship on cancer survival [35, 36]. These studies somewhat corroborated the findings of this particular study on peer mentorship, though with variations. While the previous studies were designed as programs and conducted at program sites with mentors who were not necessarily survivors, the current study was performed in the natural environment of a CSG, created and executed by the patients, for patients. Based on our evidence, peer mentorship represents a novel supportive care approach where patients guide other patients through complex care systems. This finding is significant for nonprofits planning to establish CSGs, facilities aiming to reduce abscondments from care, and policymakers striving to enhance cancer survivorship. We argue that peer mentorship should be an essential component of every CSG and survivorship care plan. While this finding is promising, it is essential to note that CSGs and facilities engaged in peer mentorship should ensure that training is provided in areas such as communication, emotional impact, patient navigation, and self-care, so that those mentoring others have the necessary tools.

### Peer support is an important part of supportive care

We found that one of the cardinal goals of the CSG is the need for patients to support each other in navigating the cancer health system. CSG members help one another through constant communication, sharing experiences of breakthroughs and survival stories, especially from survivors, to help those who are newly diagnosed gain strength in facing the cancer care process with confidence in their survival. Cancer survivors who are CSG members have received various forms of assistance from one another, including financial support and friendships that help keep their hopes alive. The study noted that CSG members have found solace through the support they receive and view the CSG as a family that genuinely cares for their well-being. Peer support is a system of giving and receiving help that is not based on psychiatric models and diagnostic criteria; rather, it focuses on understanding another’s situation and empathetically using their experience to form a connection and share support [37]. When a cancer patient meets another patient, their connection is profound, and their bond is usually strong. This is because many patients have navigated cancer care alone and coped with pain and the complexities of the weak health system with little or no support [38]. In this CSG, our findings revealed that participants provided encouragement and motivation to one another regarding their treatment experiences, which are instrumental in their survivorship. It is one thing to navigate the complex cancer healthcare system, but it is another to go through it alone. The burden that cancer patients bear is made lighter through the availability of peer support systems that not only recognize the enormity of the burden but also empathize.

The literature on the usefulness of peer support for patients with chronic health conditions is extensive. Studies from various parts of the world support the relevance of CSGs. For instance, CSGs help build relationships and foster healthy social interactions among Aboriginal females with cancer [39], improve emotional and psychological well-being in breast cancer patients in the Netherlands [40], create connections and community among patients living with metastatic breast cancer in Australia [41], and reduce distress, anxiety, and depressive symptoms in adolescents and young adults with cancer in China [42]. In Africa, Belete et al. [43] study shows that CSG influences treatment decision-making in Ethiopian cancer patients, enhances positive family relationships, and provides a safe space for patients in South Africa [44]. Our findings align with the above studies. We argue that peer support is a crucial part of supportive care; therefore, clinical oncologists and other oncology professionals should strive to connect cancer patients with fellow patients for peer support. Oncology centers of excellence across Nigeria should establish cancer support groups to ensure that patients are engaged beyond treatment.

Although peer support and mentorship are often used interchangeably, our findings show they serve complementary but distinct roles. In oncology, peer support emphasizes mutual understanding and emotional reassurance, enabling patients to reduce isolation and better cope with the immediate challenges they face. Peer mentorship is more goal-oriented, providing guidance, role modeling, and strategies for managing care and maintaining self-management. These differences are important: support improves emotional well-being and short-term adjustment, while mentorship encourages patient activation, promotes lasting behavior change, and enhances the use of health services. Recognizing these roles clarifies their contributions to patient outcomes and suggests that health systems should integrate both to improve care continuity and efficiency. While patients’ meetings foster bonds and peer support, it is also important to acknowledge the complexities of human connection and individual differences within the CSG. Potential challenges include differing coping styles, emotional transference, and the effects of recurrence or the death of a peer supporter. This highlights the importance of training and structured facilitation to manage these complexities.

### Cancer support group propels patient advocacy

Another key finding is the participants’ report on how the CSG creates an environment for patient advocacy and emphasizes that engaging patients to share their stories in leading advocacy is critical to successful policy advocacy and government engagement. In life experiences, particularly in healthcare, no one tells the story better than the person who is the primary sufferer of a health condition. This perspective is embedded in narrative medicine, which posits that every patient has a story and requires physicians who listen, give them a voice, understand, and accompany them through illness [45]. The study findings reveal that patient advocacy is demonstrated in two forms: self-advocacy and advocacy for others. Self-advocacy relates to when cancer patients have the ability to speak out for better care and treatment for themselves. In contrast, advocacy for others focuses on speaking out for policy changes regarding accessibility, affordability, and availability of care for other patients.

In both strands of patient advocacy, the personal narrative of a single cancer patient or survivor holds far more weight than that of a thousand non-cancer patients. This distinction makes our findings unique, as the CSG has played a vital role in advancing impactful patient advocacy to various government ministries and institutions in Nigeria. For example, in 2016, when Project PINK BLUE was advocating for the establishment of the National Institute for Cancer Research and Treatment (NICRAT) in the Senate, the campaigns were driven by patients’ stories. In 2017, they also drew on patients’ narratives to advocate for the National Health Insurance Scheme (now Agency) to include cancer treatment in its coverage [46, 47]. By 2019, the Scheme expanded its coverage to include cancer. Similarly, in 2019, as the Healthcare Federation of Nigeria and other partners advocated for the establishment of the Nigerian Cancer Health Fund (CHF) before Prof. Isaac F. Adewole, Minister of Health, a patient from Project PINK BLUE spoke at the meetings, sharing her personal experience with financial toxicity from cancer to motivate the government to budget for the CHF. The use of patient experiences in cancer advocacy is well documented and should be replicated by nonprofits planning for cancer policy changes [48, 49].

In other settings, reports about the effectiveness of patient advocacy in promoting cancer care, survivorship, and policy changes are plentiful [13, 50–53]. Our findings carry significant implications for cancer treatment and survivorship, especially in low- and middle-income countries where patient advocacy, voices, and government-supported care are still emerging. The implication is that clinicians, nonprofit leaders, and other relevant partners who aim to drive policy advocacy in cancer control should engage patients to utilize their experiences in demystifying the need for policy changes.

### What happens at cancer support groups

Our findings explored the various activities that occur at CSG meetings, including facilitators, barriers, benefits, and optimism within the CSG. First, patient education is a major activity emphasized by most participants. They recounted several instances of learning one or two new things during CSG meetings, which helped them enhance their understanding of the disease and their approach to it. Through patient education, cancer survivors reported acquiring self-care skills, dietary knowledge, basic exercises, and improved mood management. Many participants expressed satisfaction with the information-sharing sessions of the CSG, noting that these sessions are always rich in knowledge, as professional facilitators (i.e., oncologists, nurses, surgeons, and psychologists) are typically invited to speak with the survivors. Both clinical and non-clinical oncology education are crucial for cancer survivorship, as they provide patients with new and improved methods of care. Stolley et al. [54] reported the importance of knowledge and skill development in cancer care for enhancing health behaviors and quality of life for cancer patients. Similarly, Mackie et al. [41] reported that patient support groups provide informational support, educating members on coping mechanisms and improving health outcomes. Our findings highlight CSG’s key role in educating and empowering members through information sharing, peer modeling, and encouragement. Members learn to manage side effects, develop effective coping strategies, and communicate effectively with healthcare providers, thereby boosting their self-efficacy. This enables survivors to provide support and enhances the group’s overall impact; see Table 3.

Second, our findings also showed that the survivors engaged in various activities at CSG meetings, including singing and dancing to celebrate their survivorship, participating in photo shoots to appreciate themselves, and sharing experiences to bond with one another, see Table 3. While these activities may seem ordinary, our findings indicate that they hold significant meaning for the survivors. These activities foster positive affect, hope, and optimism. Fontesse et al.’s [55] meta-analysis reveals that positive affect is associated with longer survival among cancer patients. Similarly, Onyedibe et al. [56] showed that positive refocusing and other adaptive cognitive emotion regulation strategies are useful in reducing cancer-related fatigue among cancer survivors. In a cross-sectional study of 565 cancer patients, Onyedibe et al. [57] argued that negative generalization and self-criticism exist among cancer patients in Nigeria, thus impacting the physical well-being of the survivors. Therefore, CSG should not only function as a venue for serious discussions but also serve as a space for hosting a variety of events that promote happiness and positive interactions, lighten emotional burdens, manage stress, and encourage humor and fun activities.

**Fig. 2:**
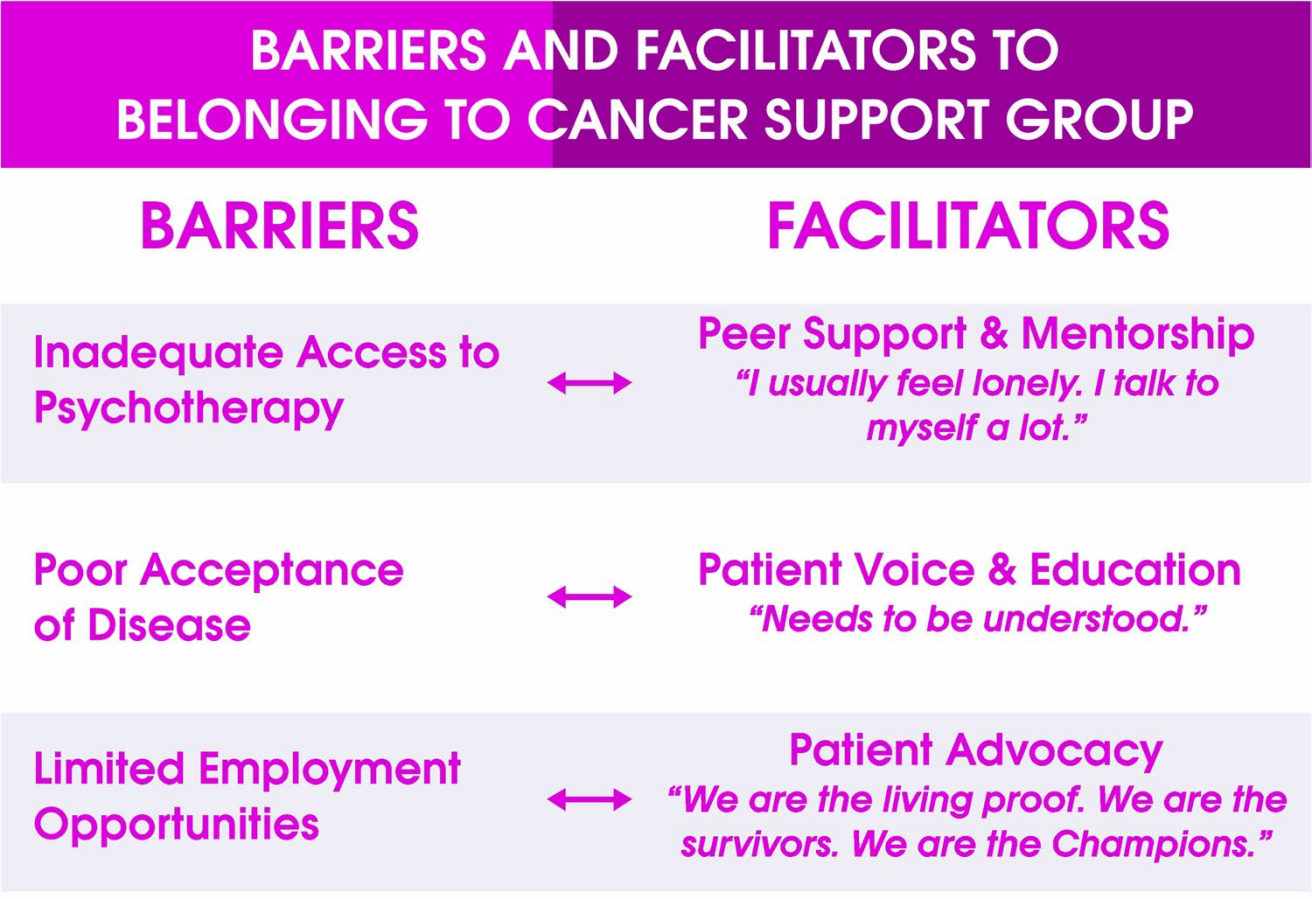
Barriers and facilitators to belonging to a cancer support group. The figure contrasts the barriers faced by participants, such as limited psychotherapy access, poor disease acceptance, and restricted employment opportunities, with the facilitators that helped mitigate these challenges. Facilitators included peer support and mentorship, patient voice and education, and patient advocacy. Patient quotes illustrate these dynamics, highlighting how group participation both counters isolation and empowers members to overcome social and emotional challenges

Third, our findings showed that the CSG uses a hybrid approach in its meetings. It hosts in-person monthly meetings and uses WhatsApp groups to connect beyond the physical meetings. While online CSG meetings are more common in high-income countries (HICs) such as France, the USA, and the UK, and are increasingly being used to connect cancer survivors [42, 58, 59], in a low- and middle-income country (LMICs) like Nigeria, in-person cancer support groups are preferred. Finally, there was a converging report on the benefits and facilitators of CSG. However, some participants expressed divergent opinions regarding the barriers and challenges faced by CSG and its members, which include a lack of funding for the CSG, unemployment among survivors, and poor acceptance of the disease (Fig. 2). Our findings imply that the creation of CSG should include plans to fund and support survivors’ reintegration into their workplace after treatment.

### Strengths, limitations, and future direction

From a methodological standpoint, the transcendental phenomenological qualitative research approach was best suited for this study. It guided the researchers in exploring the essence of experiences with CSG among survivors while setting aside biases and assumptions. The use of semi-structured interviews facilitated the collection of rich and robust data while employing both inductive and deductive thematic analysis to effectively organize themes and categories and preserve the phenomenological depth of the experiences, see Table 4. On this basis, participants’ voices (i.e., quotes/excerpts) were maintained throughout the data analysis, with minimal interpretation from the researchers. Despite its strengths, data collection from a single CSG and only 14 participants is a significant limitation; therefore, the interpretation of these results should be approached with caution. It is important to note that these findings are hypothesis-generating and would require confirmation in a more diverse setting. Most participants were breast cancer survivors and female, with only one participant being a male Hodgkin lymphoma survivor. Including male breast cancer survivors and other male cancer patients may offer a more nuanced understanding of data on CSGs. While we know that in our context, participant recruitment through the CSG leaders was useful since they know and trust them, we acknowledge that this recruitment strategy may have introduced selection bias. Future studies can achieve this by using stratified purposive sampling with predefined quotas for male participants and for major diagnostic groups beyond breast cancer, including men’s or mixed-diagnosis support groups. Analytically, comparative approaches (such as cross-case matrices by sex and cancer type) can uncover similarities and differences in CSG processes. These steps will help capture a wider range of CSG experiences and enhance the transferability of conclusions across diagnoses and sexes. Future studies should also involve additional CSGs, compare different CSGs, recruit more male participants, and consider the use of randomized controlled trials and longitudinal research designs to clarify the role of CSGs in the quality of life of cancer patients. Oncology researchers should also explore the experiences of cancer survivors with unemployment and the workplace during and after treatment in Nigeria.

## Conclusion

**Fig. 3:**
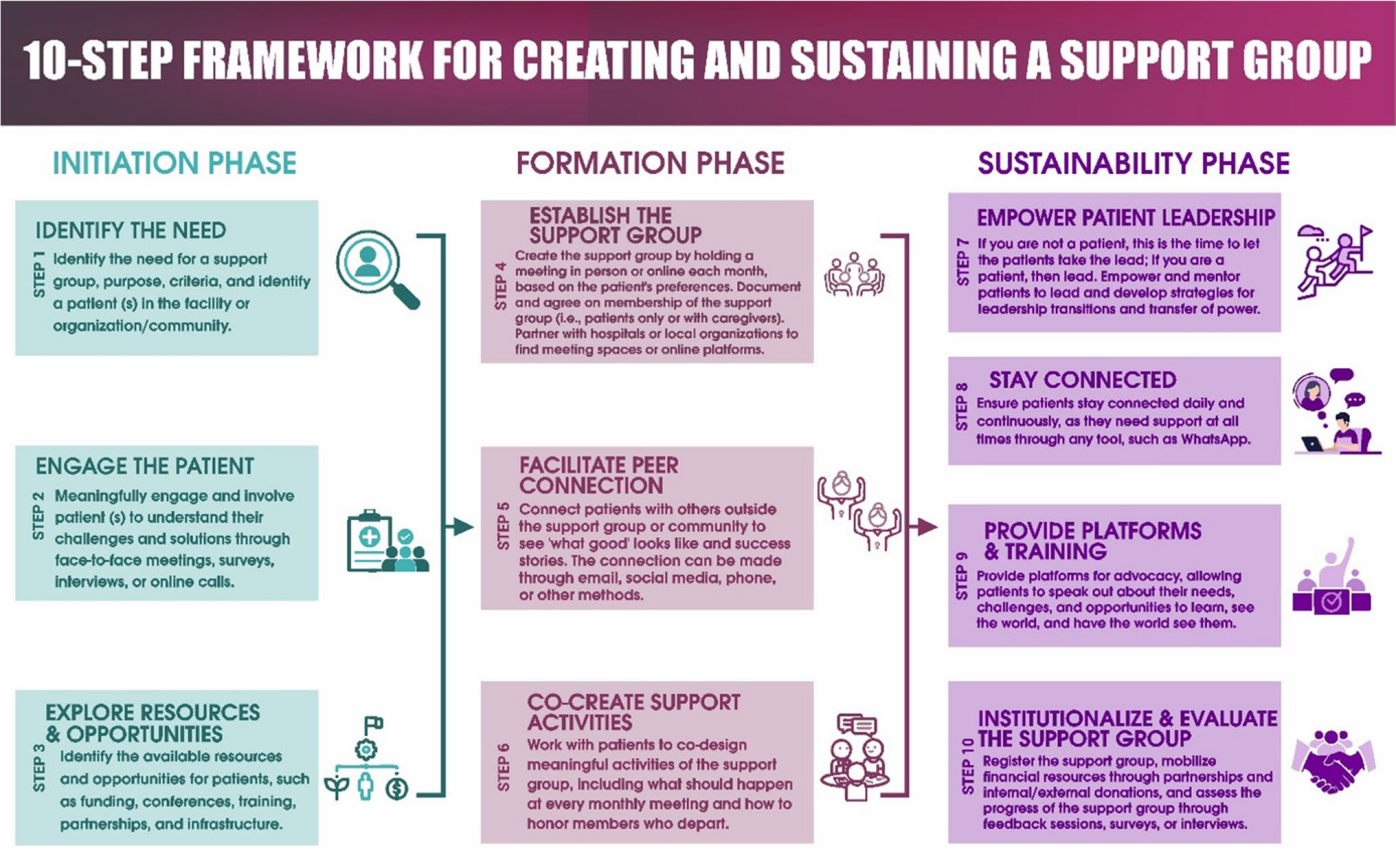
The ten-step framework for creating and sustaining a support group was developed

The study’s findings suggest that CSGs are promising interventions that warrant pilot implementation to enhance cancer survivorship in Nigeria. The study results emphasize the critical importance of CSG. The new Nigeria National Strategic Cancer Control Plan should prioritize and allocate a budget for supportive care. NICRAT should consider spearheading the campaign for all Oncology Centers of Excellence (OCE) to establish cancer support groups in their facilities; see Fig. 3 for the Ten-Step Framework for Creating and Sustaining a Support Group. The chief medical directors of the OCEs and heads of oncology units should consider collaboration with patients and patient advocacy groups to develop culturally relevant CSGs in their regions. While the Project PINK BLUE model with ABC-SG and NePICiN is useful, OCE should expand on it and ensure cultural attributes, such as languages and what is meaningful to the patients in that location, are included in the activities of the CSG. In Africa, “we feeling,” collective identity and solidarity are emphasized, often described through philosophical frameworks like “Igwebuike” and “Ubuntu,” which highlight interdependence and mutual support [60, 61]. In this context, CSGs can serve as valuable platforms that tap into patients’ internal strengths and shared experiences to foster positive outcomes. Instead of proposing a single cultural model, we emphasize that CSGs should draw on such principles in ways that are appropriate to each community. Therefore, CSGs create meaningful experiences in the cancer care journey by embodying patient-led support, guided by cultural relevance and solidarity. On this premise, we urge the African Organization for Research and Training in Cancer (AORTIC) and many other regional and national cancer bodies to promote the establishment of CSGs and supportive care across Africa. If Nigeria, Africa, and many LMICs want to make progress in their cancer control efforts, then meaningful engagement, culturally sensitive support, and collaboration with cancer patients must be prioritized.

## Supplementary Information

Below is the link to the electronic supplementary material.

ESM 1 (DOCX 536 KB)

## Data Availability

The datasets generated during and/or analysed during the current study are available from the corresponding author on reasonable request.
