# Supplementary material for: “We are champions”: an investigation on how cancer support group propels patient advocacy, voices, education, mentorship, and support
Source: Support Care Cancer. 2025 Dec 6;34(1):11. doi: 10.1007/s00520-025-10203-7 (PMC12681469; doi:10.1007/s00520-025-10203-7)
Supplement: Supplementary file 1 — (DOCX 536 KB) [file 520_2025_10203_MOESM1_ESM.docx]

**Online Supplementary Information**

**sTable 1: Consolidated criteria for reporting qualitative studies (COREQ): 32-item checklist (Tong et al., 2007)**

| **No** | **Item** | **Guide questions/descriptions** | **Checkmark (**✓) = **Done**, (X) = **Not done in this current study/Page Number** |
| --- | --- | --- | --- |
| **Domain 1: Research team and reflexivity** | | | |
| Personal Characteristics | | | |
| 1. | Interviewer/  facilitator | Which author/s conducted the interview or focus group? | (✓) |
| 2. | Credentials | What were the researcher's credentials? *E.g. PhD, MD* | (✓) |
| 3. | Occupation | What was their occupation at the time of the study? | (✓) |
| 4. | Gender | Was the researcher male or female? | (✓) |
| 5. | Experience and training | What experience or training did the researcher have? | (✓) |
| Relationship with participants | | | |
| 6. | Relationship established | Was a relationship established prior to study commencement? | (✓) |
| 7. | Participant knowledge of the interviewer | What did the participants know about the researcher? e*.g. personal goals, reasons for doing the research* | (✓) |
| 8. | Interviewer characteristics | What characteristics were reported about the interviewer/facilitator? e.g. *Bias, assumptions, reasons and interests in the research topic* | (✓) |
| **Domain 2: study design** | | | |
| Theoretical framework | | | |
| 9. | Methodological orientation and Theory | What methodological orientation was stated to underpin the study? *e.g. grounded theory, discourse analysis, ethnography, phenomenology, content analysis* | (✓) |
| Participant selection | | | |
| 10. | Sampling | How were participants selected? *e.g. purposive, convenience, consecutive, snowball* | (✓) |
| 11. | Method of approach | How were participants approached? e*.g. face-to-face, telephone, mail, email* | (✓) |
| 12. | Sample size | How many participants were in the study? | (✓) |
| 13. | Non-participation | How many people refused to participate or dropped out? Reasons? | (✓) |
| Setting | | | |
| 14. | Setting of data collection | Where was the data collected? e*.g. home, clinic, workplace* | (✓) |
| 15. | Presence of non-participants | Was anyone else present besides the participants and researchers? | (✓) |
| 16. | Description of sample | What are the important characteristics of the sample? *e.g. demographic data, date* | (✓) |
| Data collection | | | |
| 17. | Interview guide | Were questions, prompts, guides provided by the authors? Was it pilot tested? | (✓) |
| 18. | Repeat interviews | Were repeat interviews carried out? If yes, how many? | (X) |
| 19. | Audio/visual recording | Did the research use audio or visual recording to collect the data? | (✓) |
| 20. | Field notes | Were field notes made during and/or after the interview or focus group? | (✓) |
| 21. | Duration | What was the duration of the interviews or focus group? | (✓) |
| 22. | Data saturation | Was data saturation discussed? | (✓) |
| 23. | Transcripts returned | Were transcripts returned to participants for comment and/or correction? | (✓) |
| **Domain 3: analysis and findings**z | | | |
| Data analysis | | | |
| 24. | Number of data coders | How many data coders coded the data? | (✓) |
| 25. | Description of the coding tree | Did authors provide a description of the coding tree? | (✓) |
| 26. | Derivation of themes | Were themes identified in advance or derived from the data? | (✓) |
| 27. | Software | What software, if applicable, was used to manage the data? | (✓) |
| 28. | Participant checking | Did participants provide feedback on the findings? | (✓) |
| Reporting | | | |
| 29. | Quotations presented | Were participant quotations presented to illustrate the themes / findings? Was each quotation identified? e*.g. participant number* | (✓) |
| 30. | Data and findings consistent | Was there consistency between the data presented and the findings? | (✓) |
| 31. | Clarity of major themes | Were major themes clearly presented in the findings? | (✓) |
| 32. | Clarity of minor themes | Is there a description of diverse cases or discussion of minor themes? | (✓) |

**sFigure 1: Results of the Expert Feedback on 10-Step Framework for Creating and Sustaining a Support Group**

**
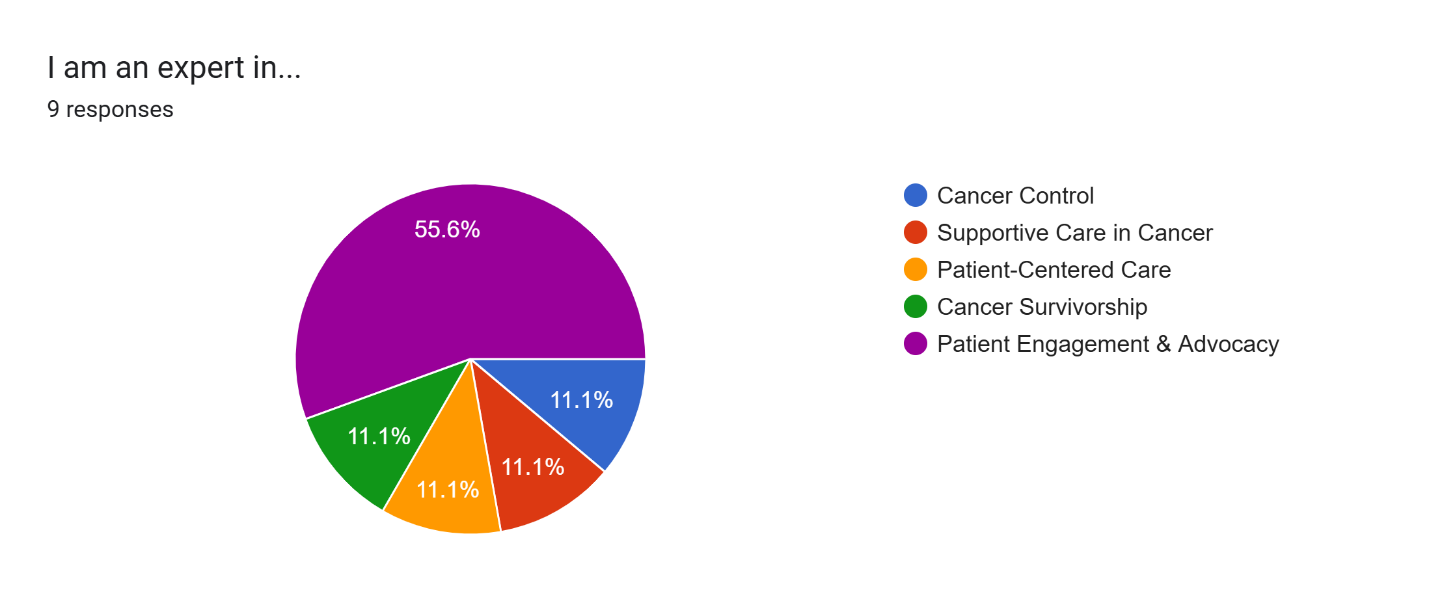

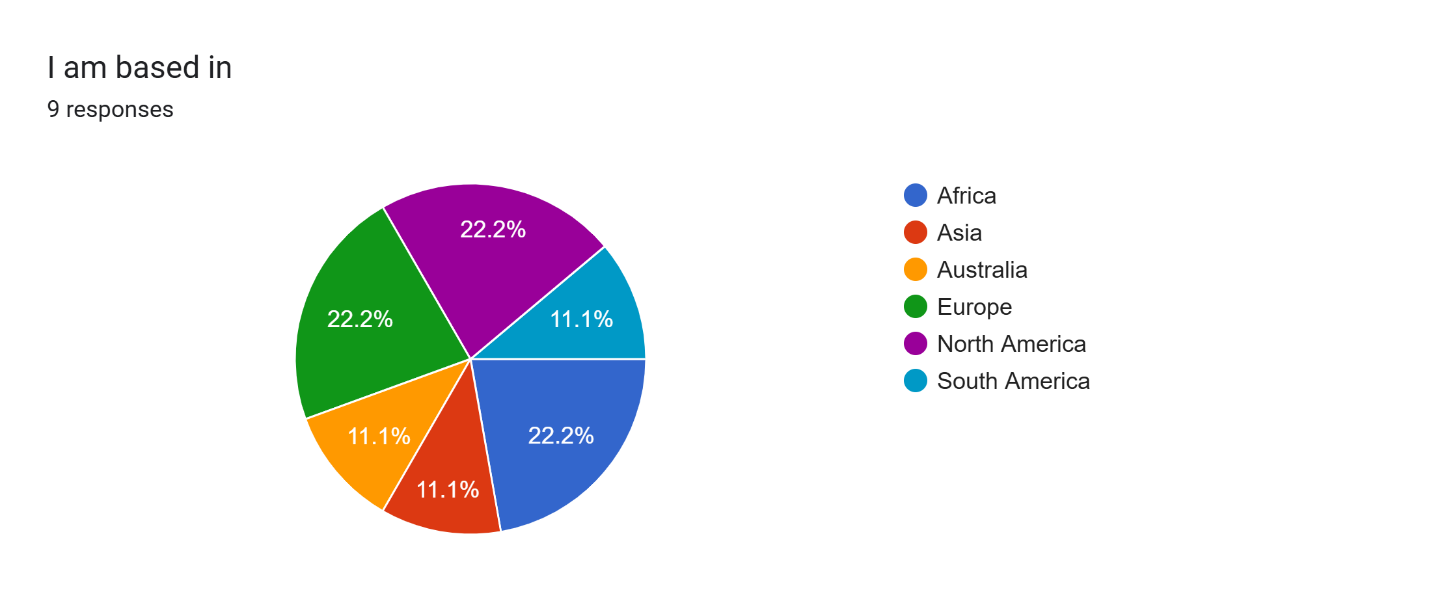
**

**
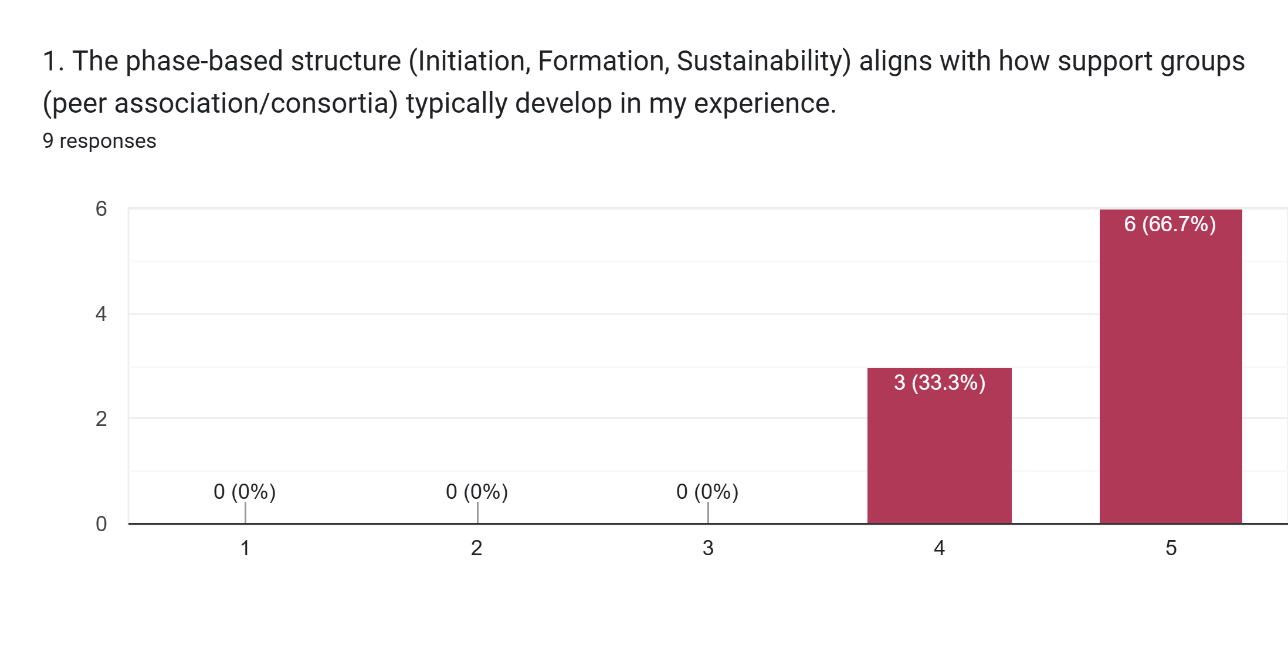

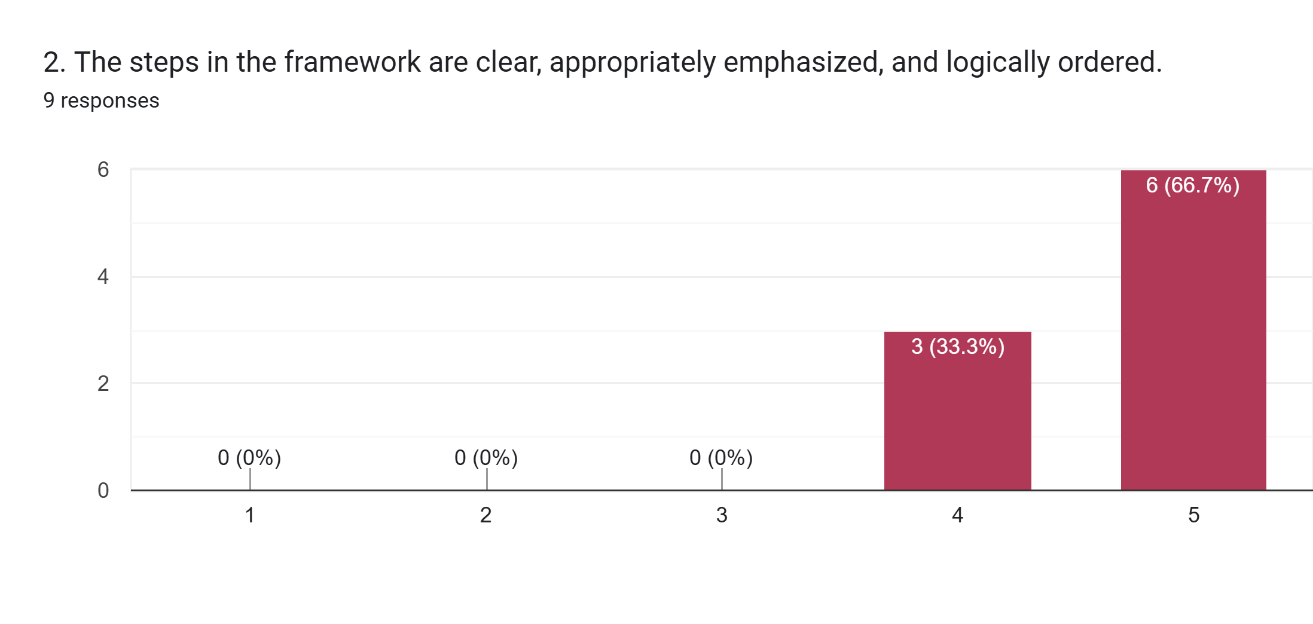

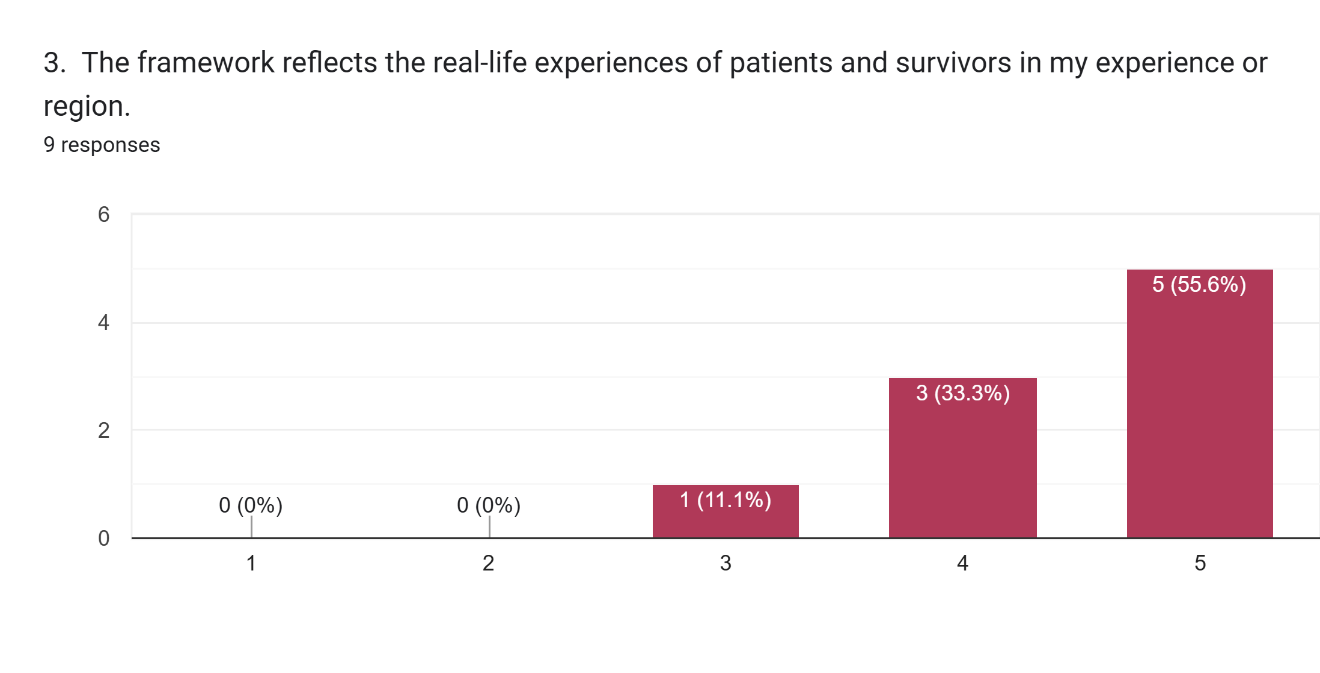
**

**
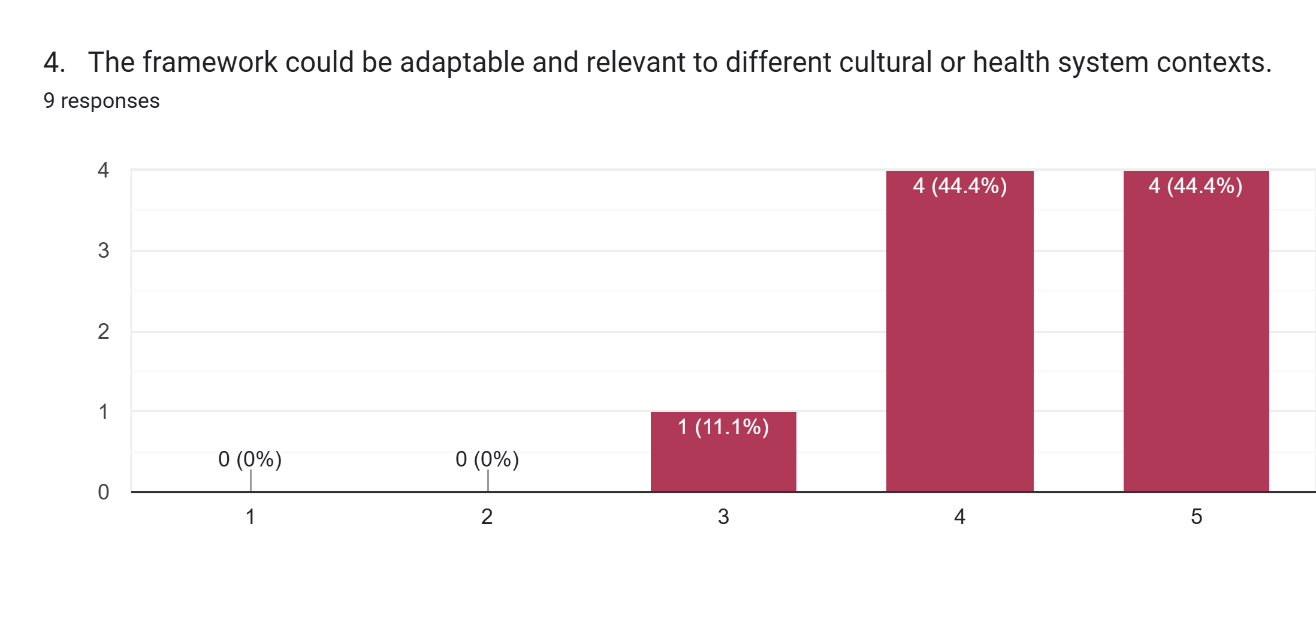

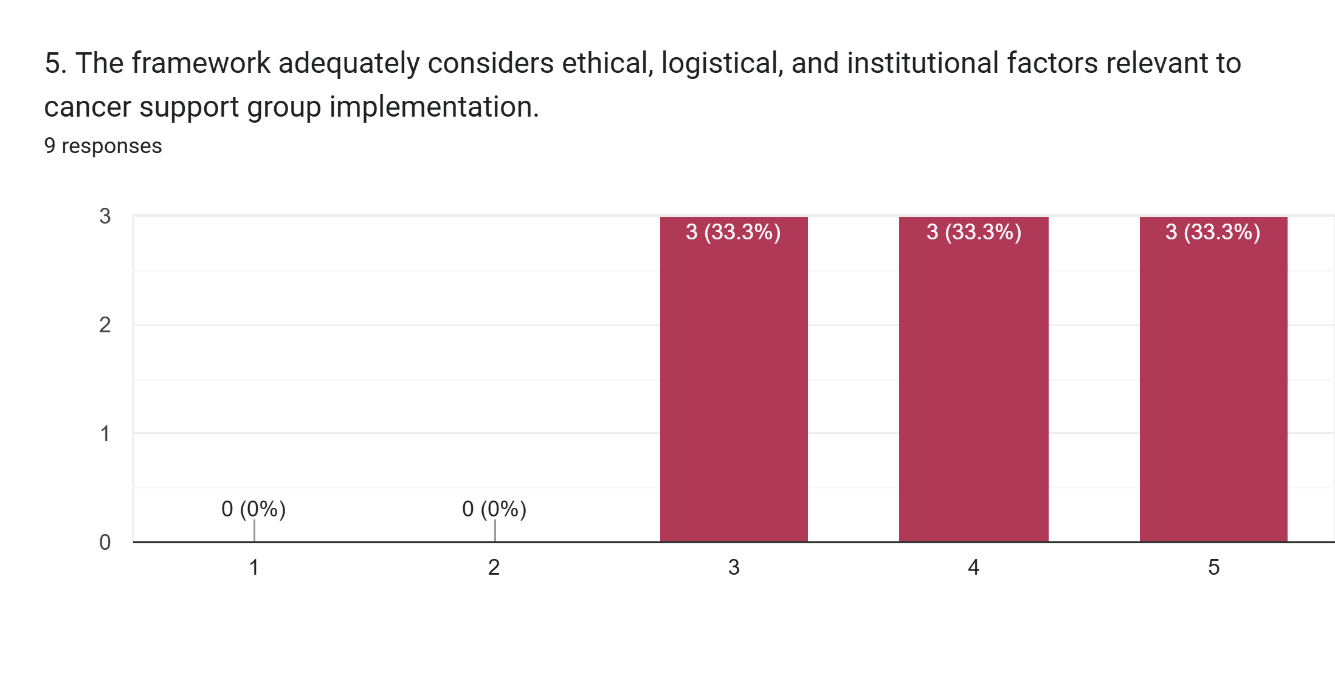

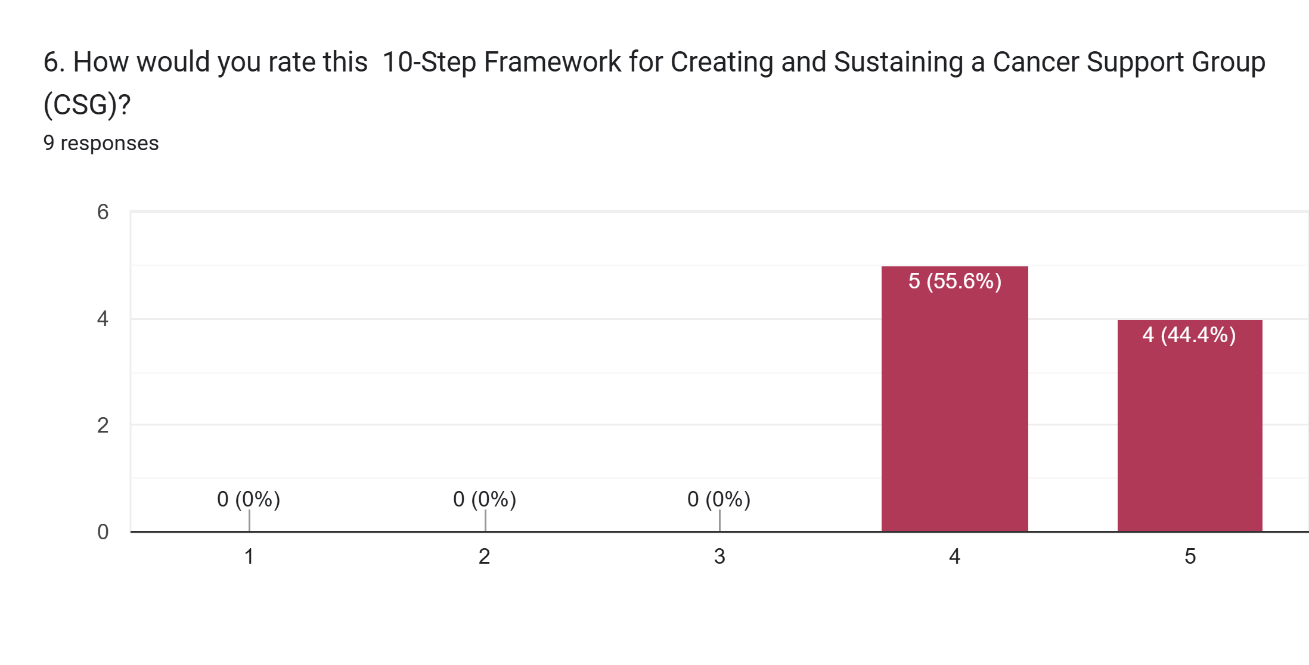
**
